# Supplementary material for: Development and characterization of a double-fluorescent HIV-1 reporter cellular model to tackle the Rev-dependent export pathway
Source: Microbiol Spectr. 2025 Feb 4;13(3):e01903-24. doi: 10.1128/spectrum.01903-24 (PMC11878058; doi:10.1128/spectrum.01903-24)
Supplement: Supplemental table and figures — Table S1; Fig. S1 to S5. [file spectrum.01903-24-s0001.docx]

**Supplementary Information**

**Supplementary Table 1. PCR and qPCR primers used in the study**

| **Primer use** | **Primer name** | **Primer sequence 5’ → 3’** |
| --- | --- | --- |
| Cloning of *mKO2* *gene* into pHIV-Intro plasmid | mKO2-Fv | TTTACTAGTATGGTTTCTGTGATCAAGCC |
|  | mKO2-Rv | TATTTCGAACTAGCTGTAGTGGGCCA |
| RT-qPCR analysis | HIV-A | CGTCTGTTGTGTGACTCTGGTAACT |
|  | HIV-B | GGATTAACTGCGAATCGTTCTAGC |
|  | HIV-C | CGAGATCCGTTCACTAATCGAATG |
|  | TAR_Fv | GTTAGACCAGATCTGAGCCT |
|  | TAR_Rv | GTGGTTCCCTAGTTAGCCA |
|  | GAPDH_Fv | CATGAGAAGTATGACAACAGC |
|  | GAPDG_Rv | AGTCCTTCCACGATACCAAAG |


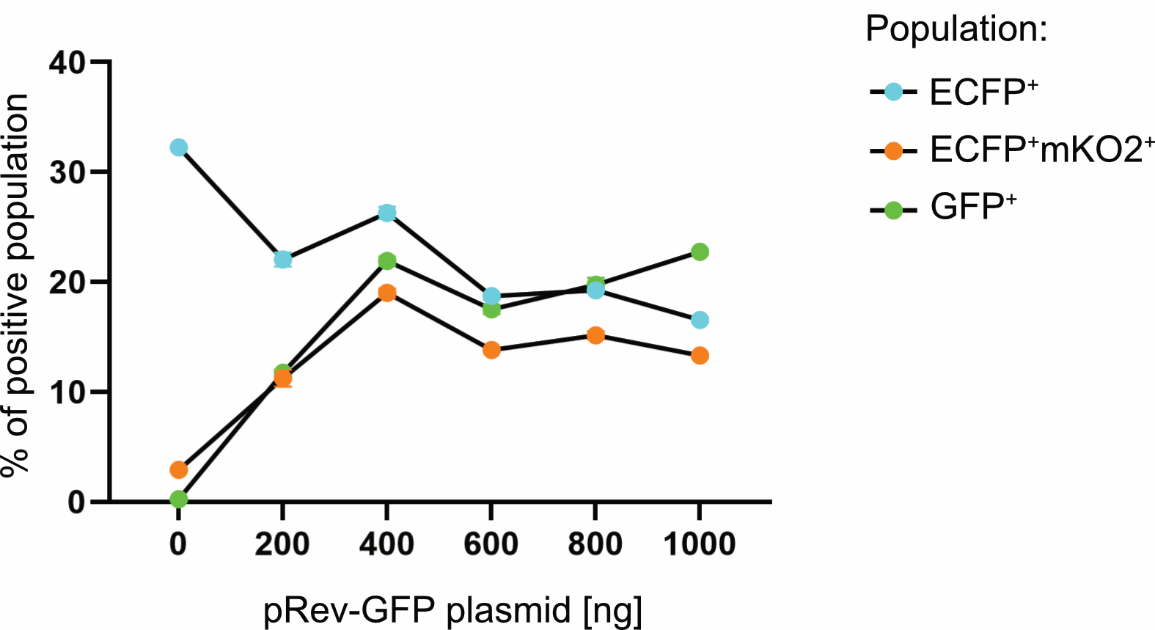


**Supplementary Figure 1.** U2OS-dual 4G10 cells were transfected with 200 ng of pTat-101 plasmid together with increasing doses of pRev-GFP plasmid and pcDNA3.1 as a carrier plasmid up to 2 µg of total transfected plasmid DNA. 24 hours after transfection, cells were subjected to flow cytometry analysis. The curves show percentages of populations: ECFP^+^mKO2^-^ (single positive, in cyan), ECFP^+^mKO2^+^ (double positive, in orange) and Rev-GFP positive cells (in green). Data comes from one biological experiment with two technical repetitions for each point.


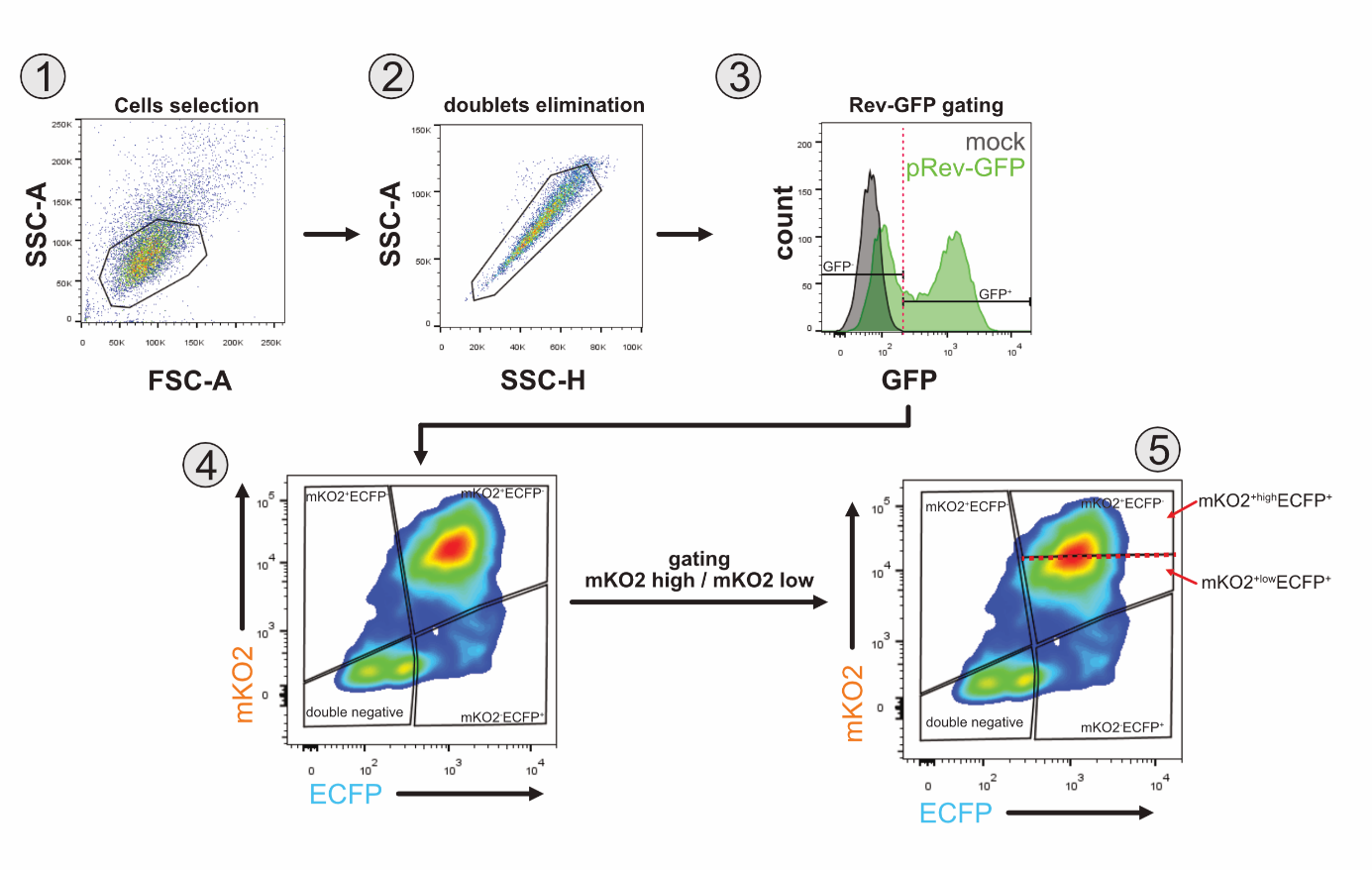


**Supplementary Figure 2.** Gating strategy for Rev-activity quantification in U2OS-dual reporter system. U2OS-dual cells were subjected to flow cytometry analysis. Step 1 – forward and side scatter plot with gate corresponding to cells; Step 2 – side scatter area and height plot with gate for single cells; Step 3 – GFP fluorescence histogram of mock (gray) and pRev-GFP transfected (green) cells, gates divide GFP negative and positive populations; Step 4 – mKO2 vs ECFP fluorescence plot of Rev-GFP transfected cells, gating indicates four populations corresponding to cells fluorescence: double negative (bottom left gate), ECFP-positive only (bottom right gate), mKO2 and ECFP double positive (top right gate), mKO2-positive only (top left gate); Step 5 – mKO2 vs ECFP fluorescence plot with gates set in the same way as in 4, with division of mKO2 and ECFP double positive population into mKO2^high^ and mKO2^low^ based on the value of MFI of mKO2 - indicated by red dotted line.


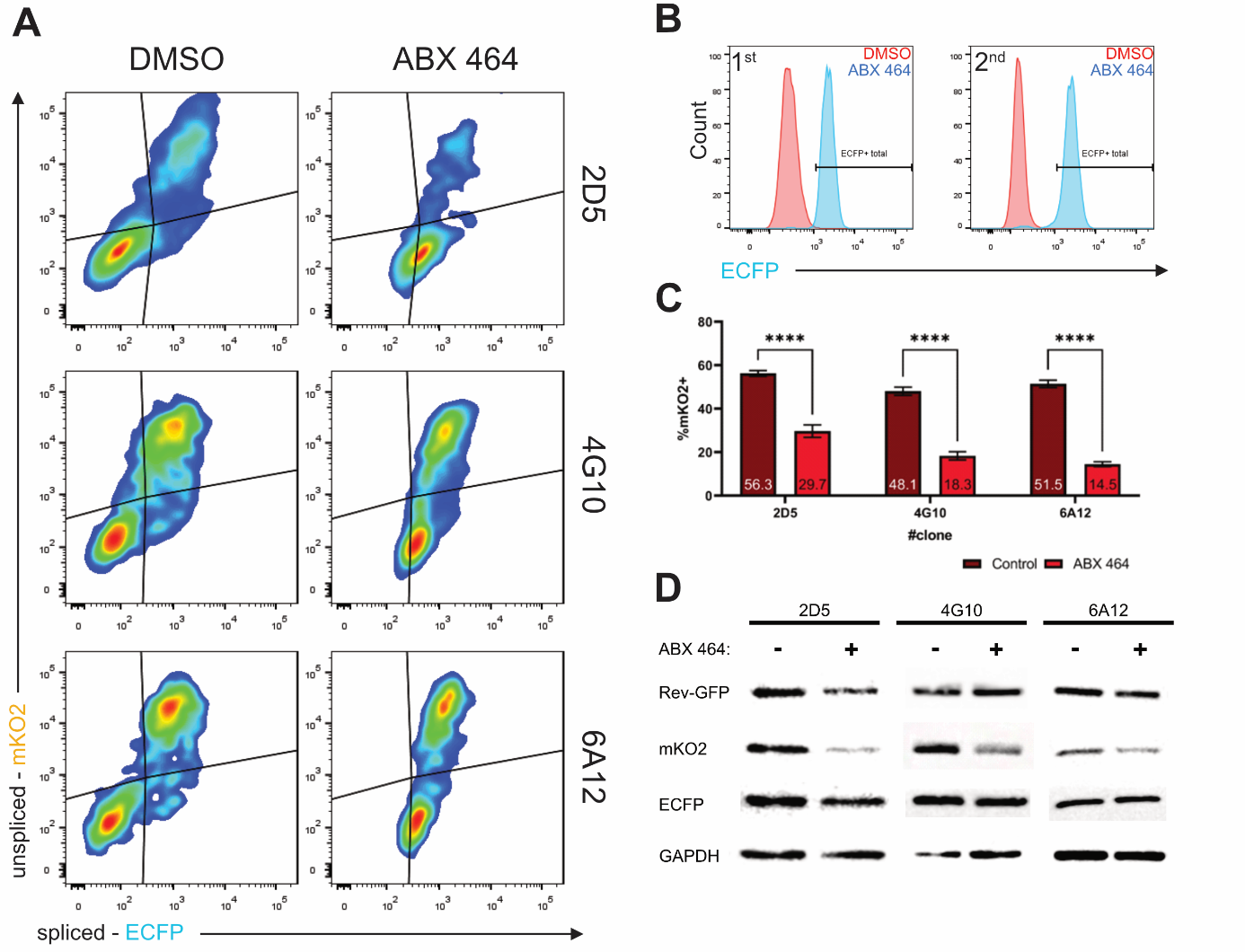


**Supplementary Figure 3.** Impact of ABX464 on Rev-dependent export. U2OS-dual clones (2D5, 4G10, 6A12) were transfected using pTat-101 and pRev-GFP plasmids followed by treatment with ABX-464 (15 µM) for 18 hours. **(A)** Representative scatterplots of mKO2 vs ECFP fluorescence in DMSO (left panels) or in ABX464 (right panels) treated U2OS-dual clones. **(B)** Flow cytometry analysis of U2OS Wild-Type cells after ABX464 treatment showing autofluorescence of the inhibitor in ECFP detector channel. Data come from two independent biological repetitions. **(C)** Flow cytometry analysis of mKO2-positive population. Data represent averages of three biological repetition (N=3), error bars indicate the standard error of mean (SEM). Statistics are performed using t-test with Welch correction, **** - p-value < 0.0001. **(D)** Western blotting analysis of the mKO2 protein levels in cell lines treated with ABX464 or mock-treated. GAPDH is a loading control.


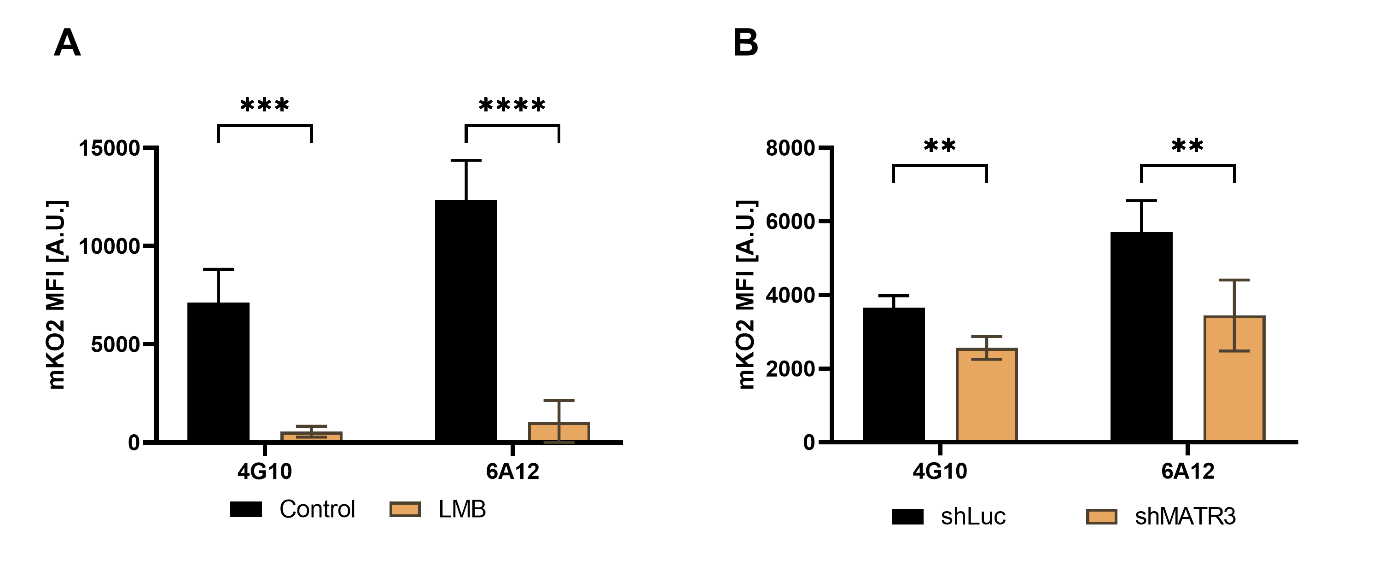


**Supplementary Figure 4.** Flow cytometry analysis of mKO2 median fluorescence intensity (MFI) in untreated or LMB treated clones. MFI measurements were conducted from Rev-GFP^+^ cells population followed by gating of ECFP^+^ cells population to minimize the effect of transfection efficiency. Data represent average of three biological repetition (N=3) and error bars indicate the standard error of mean (SEM). Statistics are performed using t-test with Welch correction, *** - p-value < 0.005, **** - p-value < 0.001.


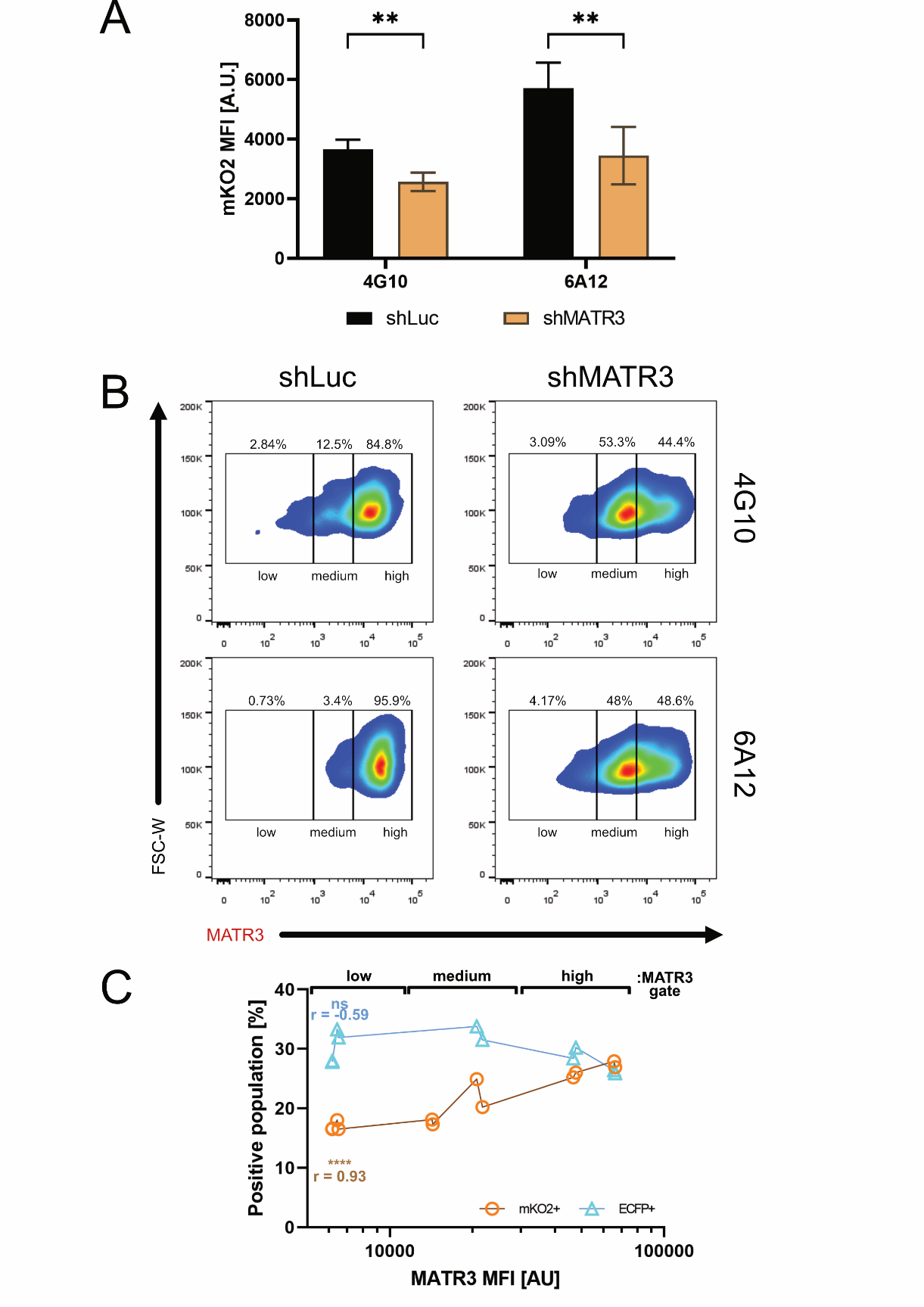


Supplementary Figure 5. **(A)** Flow cytometry analysis of mKO2 median fluorescence intensity (MFI) in shLuc and shMATR3 transduced clones. MFI measurements were conducted from Rev-GFP^+^ cells population followed by gating of ECFP^+^ cells population to minimize the effect of transfection efficiency. Data represent average of three biological repetition (N=3) and error bars indicate the standard error of mean (SEM). Statistics are performed using t-test with Welch correction, ** - p-value <0.01. (**B**) Flow cytometry analysis of MATR3+ cells. U2OS-dual clones (4G10 and 6A12) were transduced with shLuc or shMATR3 lentiviral vectors, followed by three days of puromycin selection and transfection with pTat-101 and pRev-GFP plasmids. After 24 hours, cells were collected and subjected to immunofluorescence staining with anti-MATR3 antibodies to assess MATR3+ cells. Three gates were defined to distinguish subpopulations of cells based on MATR3 fluorescence intensity (MATR3^high^, MATR3^medium^, and MATR3^low^) in shLuc-treated samples (**C**) Correlation analysis between MATR3 levels and frequency of mKO2+ and ECFP+ cells. The shMATR3-transduced 6A12 clone from panel **B** was subjected to correlation analysis to evaluate the relationship between the frequency of either mKO2+ or ECFP+ cells (derived from the three subpopulations gated in panel **B**) and MATR3 levels. Statistical analysis from two independent biological replicates (N = 2) was performed using Pearson’s correlation. The correlation coefficient (R) and p-value were as follows: mKO2 (R = 0.9281, p < 0.0001) and ECFP (R = -0.5886, p = 0.0734).
